# LncRNA *LINC00240* suppresses invasion and migration in non-small cell lung cancer by sponging *miR-7-5p*

**DOI:** 10.1186/s12885-020-07755-8

**Published:** 2021-01-09

**Authors:** Gwan Woo Ku, Yujin Kang, Seong-Lan Yu, Joonghoon Park, Sejin Park, In Beom Jeong, Min Woong Kang, Ji Woong Son, Jaeku Kang

**Affiliations:** 1 Department of Thoracic Surgery, Konyang University Hospital, Daejeon, 35365 Republic of Korea; 2 Priority Research Center, Myunggok Medical Research Institute, College of Medicine, Konyang University, Daejeon, 35365 Republic of Korea; 3 Graduate School of International Agricultural Technology and Institute of GreenBio Science and Technology, Seoul National University, Pyeongchang, 25354 Republic of Korea; 4 Department of Internal Medicine, Konyang University Hospital, Daejeon, Republic of Korea; 5 Department of Thoracic and Cardiovascular Surgery, School of Medicine, Chungnam National University, Daejeon, 35015 Republic of Korea; 6 Department of Pharmacology, College of Medicine, Konyang University, Daejeon, 35365 Republic of Korea

**Keywords:** LINC00240, miRNA-7, Non-small cell lung cancer, EGFR

## Abstract

**Background:**

lncRNAs have important roles in regulating cancer biology. Accumulating evidence has established a link between the dysregulation of lncRNAs and microRNA in cancer progression. In previous studies, *miR-7-5p* has been found to be significantly down-regulated in mesenchymal-like lung cancer cell lines and directly regulated *EGFR*. In this work, we investigated the lncRNA partner of *miR-7-5p* in the progression of lung cancer.

**Methods:**

We investigated the expression of *miR-7-5p* and the lncRNA after transfection with an *miR-7-5p* mimics using a microarray. The microarray results were validated using quantitative real time-polymerase Chain Reaction (qRT-PCR). The regulatory effects of lncRNA on *miR-7-5p* and its target were evaluated by changes in the expression of *miR-7-5p* after transfection with siRNAs for lncRNA and the synthesis of full-length lncRNA. The effect of *miR-7-5p* on lncRNA and the miRNA target was evaluated after transfection with miRNA mimic and inhibitor. The role of lncRNA in cancer progression was determined using invasion and migration assays. The level of lncRNA and EGFR in lung cancer and normal lung tissue was analyzed using TCGA data.

**Results:**

We found that *LINC00240* was downregulated in lung cancer cell line after *miR-7-5p* transfection with an *miR-7-5p* mimic. Further investigations revealed that the knockdown of *LINC00240* induced the overexpression of *miR-7-5p*. The overexpression of *miR-7-5p* diminished cancer invasion and migration. The *EGFR* expression was down regulated after siRNA treatment for *LINC00240*. Silencing *LINC00240* suppressed the invasion and migration of lung cancer cells, whereas *LINC00240* overexpression exerted the opposite effect. The lower expression of *LINC00240* in squamous lung cancer was analyzed using TCGA data.

**Conclusions:**

Taken together, *LINC00240* acted as a sponge for *miR-7-5p* and induced the overexpression of *EGFR*. *LINC00240* may represent a potential target for the treatment of lung cancer.

**Supplementary Information:**

The online version contains supplementary material available at 10.1186/s12885-020-07755-8.

## Background

Lung cancer is a major cause of cancer deaths from, and it’ s incidence has significantly increased in the past decades. Although there has been great progress in diagnostic methods, surgical techniques, and new chemotherapy regimens in the last few decades, the 5-year survival rate for patients with non-small cell lung cancer (NSCLC) remains poor [1]. Therefore, numerous studies involved in the carcinogenesis and the progression of NSCLC have been conducted and new effective therapeutic targets for NSCLC have been reported.

Long non-coding RNAs (lncRNAs) are a heterogeneous group of non-coding transcripts more than 200 nucleotides in length, which affect various processes through a myriad of molecular functions, including the modulation of transcriptional profiles, protein activity control, complex structural or organizational roles, alteration of RNA processing events, and small RNAs precursors [2]. Studies have claimed that lncRNAs are at the center of various physiological and pathological processes associated with cell cycle progression, apoptosis during cellular development and differentiation, as well as immune system [3]. They play important roles in chromatin remodeling, transcriptional repression and post-transcriptional regulation. It is now widely understood that lncRNAs serve as signals of specific cellular states or readouts of active cellular programs. The molecular mechanisms of lncRNAs are traditionally classified into four archetypes: signals, decoys, guides and scaffolds [4]. Several lncRNAs possess characteristics from multiple archetypes that, in combination, are critical to their eventual biological function. Recent studies revealed that some lncRNAs assumed the role of molecular sponges, a behavior akin to that of competitive endogenous RNAs (ceRNAs), through miRNAs binding sites, and subsequently repressed their inhibitory effect on their natural targets Though not fully elucidated to date, some consistent threads of evidence have emerged on the dysregulation of lncRNAs’ principal role with regard to tumorigenesis and tumor progression in various cancer types [5, 6]. A number of lncRNAs have been implicated in NSCLC initiation and development, which demonstrate their potential value as diagnostic or prognostic biomarkers and therapeutic targets for NSCLC [7, 8].

miRNAs are single-stranded, small (18–24 nt) noncoding RNA molecules that directly interact with target mRNAs; a function that serves to affect tumor progression and development [9]. Mature miRNAs regulate their target genes through partial sequence complementarity to the 3′ untranslated region (UTR) of the target genes, thereby resulting in mRNA degradation or/and translational repression [10, 11]. Competing endogenous RNAs (ceRNAs) are transcripts that can regulate each other at post-transcription level by competing for shared miRNAs. CeRNA networks link the function of protein-coding mRNAs with that of non-coding RNAs such as microRNA, long non-coding RNA, pseudogenic RNA and circular RNA. Various lncRNAs molecular mechanisms are involved in cancer progression and metastasis. Accumulating evidence indicates that the sponging ability of miRNA results in the degradation or retention of targeted genes. Crosstalk between lncRNAs and their associated miRNAs will provide valuable insights into cancer biology and therapeutic targets for NSCLC. The expression of *miR-7-5p,* a highly controlled 23-nucleotide miRNA, appears to be predominant in a limited number of organs and systems such as brain, spleen, and pancreas. Reduced levels of *miR-7-5p have been associated with* cancer growth and metastasis. Given the special role of coordinate downregulation of both direct (epidermal growth factor receptor) and indirect (phospho-Akt) growth-promoting targets, *miR-7-5p* performs a tumor-suppressor function, which ultimately serves to impede the development of tumor in vitro and vivo. In addition, *miR-7-5p* can significantly enhance the overall sensitivity of treatment-resistant cancer cells to therapeutics and hamper metastasis dissemination [12].

We investigated the expression of *miR-7-5p* and the lncRNA profile after transfection with a miRNA-7*-5p* mimic using a microarray. The microarray results were validated using quantitative real time-polymerase chain reaction (qRT-PCR). The regulatory effects of lncRNA on *miR-7-5p* and its target were evaluated. The effect of *miR-7-5p* on lncRNA and the miRNA target was evaluated and the role of lncRNA in cancer progression was determined. The role of LINC00240 has been reported in esophageal squamous cell carcinoma (ESCC) and cervical cancer progression. In the present study, we searched for *miR-7-5p* and related LINC00240 in lung cancer.

## Methods

### Cell culture

Human non-small cell lung cancer cell lines (A549, H23, H226, H292, H358, H460, H522, H1299, Hcc95, Hcc827, and Hcc1438) were obtained from the Korean Cell Line Bank (Seoul, Korea). The cells were cultured in RPMI-1640 medium (Hyclone, Logan, UT, USA) or DMEM/HIGH GLUCOSE (A549; Hyclone) supplemented with 10% fetal bovine serum (FBS; Gibco, Life Technologies, Grand Island, NY, USA) and 1% penicillin/streptomycin solution (Hyclone). The Hcc95 cells were supplemented HEPS. The cells were maintained at 37 °C in a humidified atmosphere containing 5% CO_2_.

### Transfection

A synthetic *hsa-miR-7-5p* mimic (Genolution, Seoul, Korea) was designed according to registered in miRBase database. miRNA inhibitor targeting *hsa-miR-7-5p* (5′-UGG AAG ACU AGU GAU UUU GUU GUU-3′) and an siRNA targeting *LINC00240* (5′-CUA CAU UUG AGC AUA GUA U-3′) were synthesized by Bioneer Co. (Bioneer, Daejeon, Korea). Cell transfection was performed using Lipofectamine RNAiMAX reagent (Invitrogen, Carlsbad, CA, USA) according to the manufacturer’s protocol. The synthesized full-length *LINC00240* sequence was subcloned into pcDNA3.1 vectors and, cell transfection was performed using a Lipofectamine 3000 transfection kit (Invitrogen) by following the manufacturer’s protocol.

### Target labeling and hybridization to microarray

We synthesized target cRNA probes and performed hybridization using Agilent’s Low RNA Input Linear Amplification kit (Agilent Technology, USA) according to the manufacturer’s instructions. Briefly, each 0.2 μg total RNA sample was mixed with T7 promoter primer mix and incubated at 65 °C for 10 min. cDNA master mix (5X First strand buffer, 0.1 M DTT, 10 mM dNTP mix, RNase-Out, and MMLV-RT) was prepared and added to the reaction mixture. The samples were incubated at 40 °C for 2 h and then the RT and dsDNA syntheses were terminated by incubating at 65 °C for 15 min.

The transcription master mix was prepared according to the manufacturer’s protocol (4X Transcription buffer, 0.1 M DTT, NTP mix, 50% PEG, RNase-Out, inorganic pyrophosphatase, T7-RNA polymerase, and cyanine 3/5-CTP). Transcription of the dsDNA was performed by adding the transcription master mix to the dsDNA reaction samples and incubating at 40 °C for 2 h. The amplified and labeled cRNA was purified on an RNase mini column (Qiagen) according to the manufacturer’s protocol. Labeled cRNA target was quantified using an ND-1000 spectrophotometer (NanoDrop Technologies, Inc., Wilmington, DE, USA).

After determining the labeling efficiency, each 825 ng of cyanine 3-labeled and cyanine 5-labeled cRNA target was mixed and the cRNA fragmentation was performed by adding a 10X blocking agent and 25X fragmentation buffer and incubating at 60 °C for 30 min. The fragmented cRNA was resuspended in 2X hybridization buffer and directly pipetted onto an assembled Agilent Human Whole Genome 60 K V3 microarray. The arrays were hybridized at 65 °C for 17 h using an Agilent Hybridization oven (Agilent Technology) as described previously [13]. The hybridized microarrays were washed according to the manufacturer’s washing protocol (Agilent Technology).

### Data acquisition and analysis

The hybridization images were analyzed by an Agilent DNA Microarray Scanner (Agilent Technology) and data quantification was performed using Agilent Feature Extraction software 9.3.2.1 (Agilent Technology) as described previously [14]. The average fluorescence intensity for each spot was calculated and the local background was subtracted. All data normalization and the selection of fold-changed genes were performed using GeneSpringGX 7.3.1 (Agilent Technology). The genes were filtered to remove flag-out genes in each experiment. Intensity-dependent normalization (LOWESS) was performed, where the ratio was reduced to the residual of the Lowess fit of the intensity vs. ratio curve. The average of the normalized ratios was calculated by dividing the average of the normalized signal channel intensity by the average of the normalized control channel intensity. Genes changed > 2.0-fold were selected and considered significant genes. The microarray result has been deposited into the Gene Expression Omnibus (GEO; GSE158940).

The functional annotation of genes was performed according to Gene OntologyTM Consortium (http://www.geneontology.org/index.shtml) by GeneSpringGX 7.3.1.

### Quantitative real-time PCR (qRT-PCR)

The total RNA was isolated from the cells using TRI Reagent (Ambion, Thermo Fisher Scientific) according to the manufacturer’s protocol. To determine the mRNA and lncRNA expression levels, cDNA was synthesized using M-MLV reverse transcriptase (Promega, Madison, WI, USA) after which qRT-PCR was performed in triplicate for the *LINC00240*, *EGFR* and *GAPDH* genes using iQ SYBR Green Supermix (BioRad Laboratories, Hercules, CA, USA), and a CFX Connect Real-Time PCR Detection System (BioRad Laboratories, Hercules) was used. The qRT-PCR conditions were, 95 °C for 3 min; 39 cycles of 95 °C for 10 s, 60 °C for 15 s, and 75 °C for 15 s. The primers used for the mRNA qRT-PCR were: *LINC00240*: forward: 5′-AGG TCA CCC ACC GGT CTG AA-3′, and reverse: 5′-TAG GCT GGG CTC AGC TGG AT-3′; *EGFR*: forward: 5′-CCA GAC TCT TTC GAT ACC CA-3′, and reverse: 5′-CTT CCT GGC TAG TCG GTG TA-3′; *GAPDH*: forward: 5′- ACA GTC AGC CGC ATC TTC TT-3′, and reverse: 5′- ACG ACC AAA TCC GTT GAC TC-3′. qRT-PCR for the miRNA for the *miR-7-5p* (Assay ID: 005723_mat) and *RNU6B* (Assay ID: 001093) expression levels was performed in triplicate using TaqMan MicroRNA Assays (Applied Biosystems, Foster City, CA, USA) by following the manufacturer’s instructions. *GAPDH* and *RNU6B* were used as internal controls for normalization, respectively.

### Invasion and migration assays

Invasion and migration assays were performed using 48-well micro-chemotaxis Boyden chambers that contained 12-μm-pore membranes (Neuroprobe, Gaithersburg, MD, USA) pre-coated with 10 μg/ml Matrigel (BD Bioscience, San Jose, CA, USA) for the invasion assay and 13 μg/ml collagen type I (Sigma Aldrich) for the migration assay as described previously [15]. The cells, H1299 (0.8 × 10^6^ cells/ml) and Hcc1438 (1.3 × 10^6^ cells/ml), were seeded in triplicate in the chambers, and incubated for 24–26 h. The resulting membranes were fixed and stained using Diff-Quik reagent (Sysmex Corporation, Kobe, Japan). The invaded and migrated cells were photographed under a light microscope, and the relative invasion and migration rates were calculated based on comparison to the negative controls.

### Luciferase assay

To investigate whether *LINC00240* directly interacted *miR-7-5p*, luciferase assay was performed. The fragment of *LINC00240* containing the *miR-7-5p* binding sites were synthesis by PCR, and these are cloned the renilla luciferase reporter site of psiCHECK2 vectors. And we confirmed successful cloning using DNA sequencing. To luciferase assay, Hcc1438 cells were transfected with psiCHECK-2 plasmid containing position s 174–179 of the *LINC00240* with or without mutations of binding site and with *miR-7-5p* mimic or negative control. At 48 h after transfection, the luciferase assays were performed in manufacturer’s protocol using Dual-Luciferase Reporter Assay System (Promega). Renilla luciferase activity was measured using a Synergy HTX microplate reader (BioTek, Winooski, VT, USA), and the results were normalized using the activity of firefly luciferase. All experiments were performed in duplicate.

### The Cancer genome atlas (TCGA) program

The TCGA dataset of lung adenocarcinoma (AC) and lung squamous cell carcinoma (SQ) was downloaded from the TGCA Data Portal (https://tcga-data.nci.nih.gov/tcga/). The dataset contained a total of 1016 RNA-seq data at level 3 (515 of AC and 501 of SQ RNA-seq data) as well as clinical data. Among them, 58 AC and 51 SQ cases had RNA-seq dataset of non-tumor pairs with complete clinical information. These cohorts were used for RNA expression analysis of *LINC00240* and *EGFR*. Gene expression was measured by the Illumina HiSeq platform and presented as FPKM values. The dataset can be used for publication without restriction or constraint according to the publishing guidelines (https://cancergenome.nih.gov/publications/ publicationguidelines).

### Statistical analysis

Each experiment was performed three times, and the data were expressed as mean ± standard deviation (SD). The results were analyzed using Student’s t-test. *P*-values of ≤ 0.05 was considered statistically significant.

## Results

### Expression levels of *LINC00240* and *EGFR* were suppressed by *miR-7-5p*

**Fig. 1:**
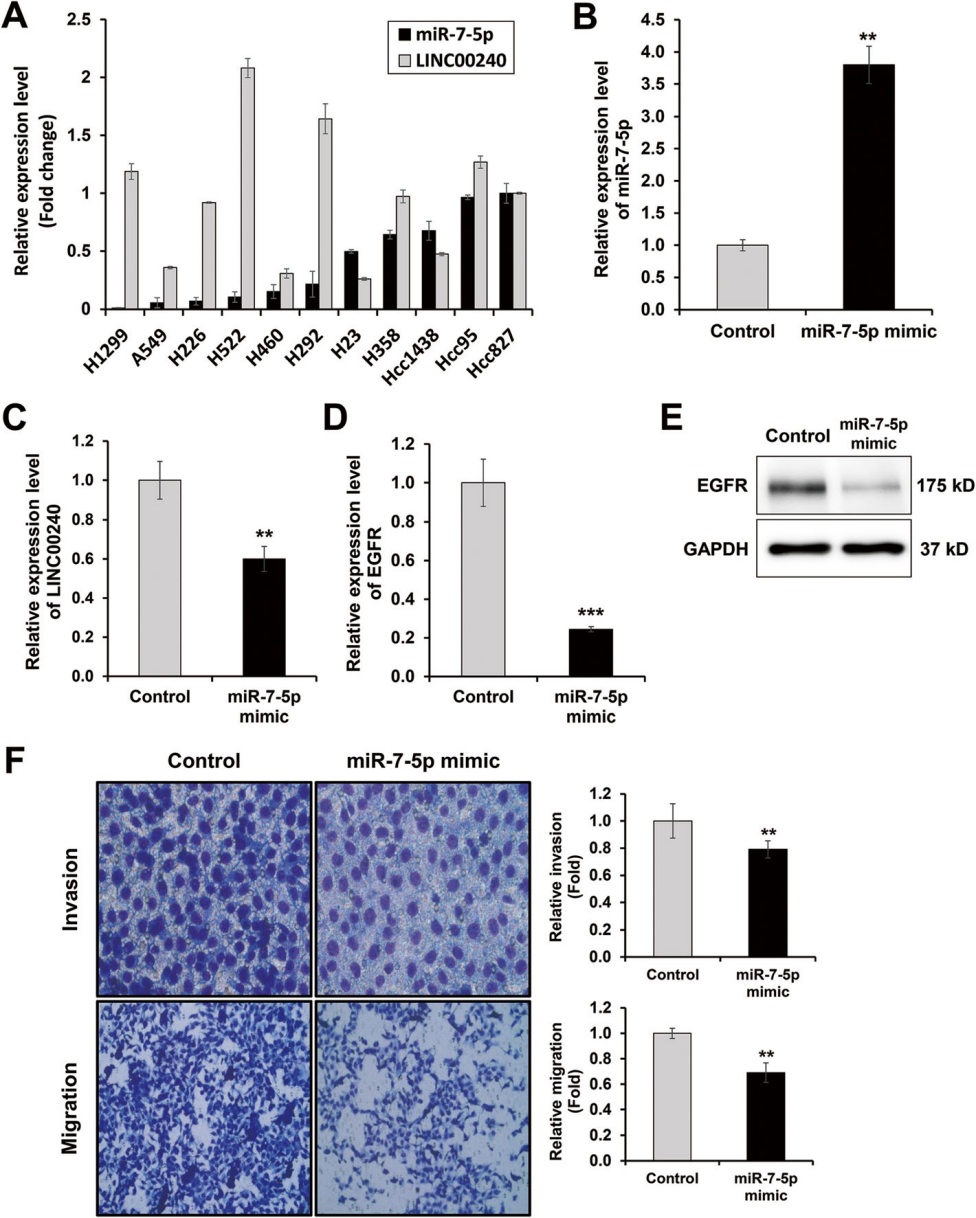
Expression level of *miR-7-5p* and *LINC00240* in NSCLC cell line, and *miR-7-5p* suppresses expression level of *LINC00240*, EGFR, cell invasion and migration in H1299. **a** Expression level of *miR-7-5p* and *LINC00240* in NSCLC cell line (A549, H23, H226, H292, H358, H460, H522, H1299, Hcc95, Hcc827 and Hcc1438). **b**-**e** Expression level of *miR-7-5p*, *LINC00240* and *EGFR* in H1299 overexpressed *miR-7-5p*. **f** *miR-7-5p* overexpression decrease cell invasion and migration in H1299. **P < 0.01 and ***P < 0.001

To identify the target genes of *miR-7-5p* known as a tumor suppressor, we first investigated its expression levels in 11 NSCLC cell lines (Fig. 1a). Among the 11 NSCLC cell lines, Hcc827 and H1299 cells showed the highest and lowest expression levels of *miR-7-5p*, respectively. The expression was elevated in the Hcc827 cells, whereas relatively low *miR-7-5p* expression levels were observed in the H1299 cell line. We performed an ectopic overexpression of *miR-7-5p* in the H1299 cells with the lowest level of *miR-7-5p*, as shown in Fig. 1a, and then we performed transcript profiling analysis to determine the genes regulated by *miR-7-5p*. Using a 2-fold change cut-off value in the transcriptome, we selected 324 genes significantly up-regulated in H1299 cells overexpressing *miR-7-5p*. Three hundred five genes were down-regulated by the overexpression of *miR-7-5p* (GSE158940, Supplementary table 1). Among them, 32 lncRNAs were up-regulated and 33 lncRNAs were down-regulated in H1299 cells overexpressing *miR-7-5p*. The top 20 regulated lncRNAs are shown in Table 1. In the differentially expressed genes, we focused on *EGFR* and *LINC00240* (lncRNA) because *EGFR* known as one of the master regulators in lung cancer is direct target of *miR-7-5p* [16]. In addition, these genes were decreased in the microarray expression data by *miR-7-5p* overexpression (supplementary table 1). We identified LINC00240 expression in 11 NSCLC cell lines and inverse relationship between *LINC00240* and *miR-7-5p* in H1299 and Hcc1438 (Fig. 1a). We confirmed the expression levels of *EGFR* and *LINC00240* using qRT-PCR to validate the microarray expression data. The expression levels of *LINC00240* and *EGFR* were down-regulated in H1299 cells overexpressing *miR-7-5p* (Fig. 1c, d). Moreover, EGFR protein expression was down-regulated by *miR-7-5p* overexpression (Fig. 1e). These data suggest that *miR-7-5p* acts as an upstream regulator of *EGFR,* and *LINC00240* is regulated by *miR-7-5p* in NSCLC.

**Table 1:** Top 20 of lncRNAs differentially expressed in *miR-7-5p* overexpressed H1299

	Fold Change	GeneSymbol	GeneName	***p***-value
**Up**	3.42	*lnc-ARHGEF5–2*	lnc-ARHGEF5–2:2	0.145
	3.08	*LINC00963*	long intergenic non-protein coding RNA 963	0.101
	2.91	*lnc-NAV3–1*	lnc-NAV3–1:10	0.122
	2.78	*lnc-RP11–688I9.2.1–1*	lnc-RP11–688I9.2.1–1:1	0.110
	2.73	*lnc-IDH2–1*	lnc-IDH2–1:1	0.119
	2.71	*lnc-HIVEP1–2*	lnc-HIVEP1–2:3	0.110
	2.67	*lnc-SMCR7–1*	lnc-SMCR7–1:1	0.126
	2.56	*LINC01111*	long intergenic non-protein coding RNA 1111	0.121
	2.51	*lnc-PLS3–2*	lnc-PLS3–2:1	0.150
	2.50	*lnc-SEC24D-1*	lnc-SEC24D-1:2	0.149
	2.43	*LINC00540*	long intergenic non-protein coding RNA 540	0.119
	2.43	*LINC01122*	long intergenic non-protein coding RNA 1122	0.115
	2.39	*lnc-SEZ6L2–1*	lnc-SEZ6L2–1:1	0.117
	2.37	*lnc-ETV3–2*	lnc-ETV3–2:2	0.119
	2.32	*lnc-PLEKHH2–2*	lnc-PLEKHH2–2:1	0.121
	2.29	*LINC00570*	long intergenic non-protein coding RNA 570	0.156
	2.28	*lnc-H2AFV-1*	lnc-H2AFV-1:1	0.171
	2.28	*lnc-ADAMTS18–1*	lnc-ADAMTS18–1:1	0.151
	2.28	*lnc-OCM-1*	lnc-OCM-1:2	0.123
	2.26	*lnc-FOXG1–6*	lnc-FOXG1–6:15	0.170
**Down**	0.19	*lnc-COL9A1–1*	lnc-COL9A1–1:1	0.082
	0.20	*lnc-FKBP2–1*	lnc-FKBP2–1:1	0.101
	0.25	*lnc-SMARCAL1–2*	lnc-SMARCAL1–2:5	0.093
	0.27	*lnc-OPN4–1*	lnc-OPN4–1:2	0.104
	0.29	*lnc-APITD1–1*	lnc-APITD1–1:2	0.106
	0.34	*lnc-FKBP2–1*	lnc-FKBP2–1:2	0.101
	0.37	*lnc-COPZ2–1*	lnc-COPZ2–1:1	0.149
	0.40	*lnc-RWDD3–6*	lnc-RWDD3–6:1	0.137
	0.44	*lnc-ANGPTL2–2*	lnc-ANGPTL2–2:3	0.151
	0.44	*lnc-SERPINC1–1*	lnc-SERPINC1–1:24	0.123
	0.44	*lnc-FAM43A-2*	lnc-FAM43A-2:1	0.154
	0.45	*lnc-OR1F1–1*	lnc-OR1F1–1:1	0.150
	0.45	*lnc-UNC93B1–2*	lnc-UNC93B1–2:1	0.140
	0.46	*lnc-DFFB-3*	lnc-DFFB-3:1	0.170
	0.47	*LINC00240*	long intergenic non-protein coding RNA 240	0.199
	0.47	*lnc-MFSD6–1*	lnc-MFSD6–1:1	0.131
	0.47	*lnc-EVX1–5*	lnc-EVX1–5:3	0.197
	0.48	*LINC01534*	long intergenic non-protein coding RNA 1534	0.160
	0.48	*LINC00999*	long intergenic non-protein coding RNA 999	0.190
	0.48	*lnc-RASA1–3*	lnc-RASA1–3:20	0.254

### *miR-7-5p* suppressed cell invasion and migration in NSCLC

**Fig. 2:**
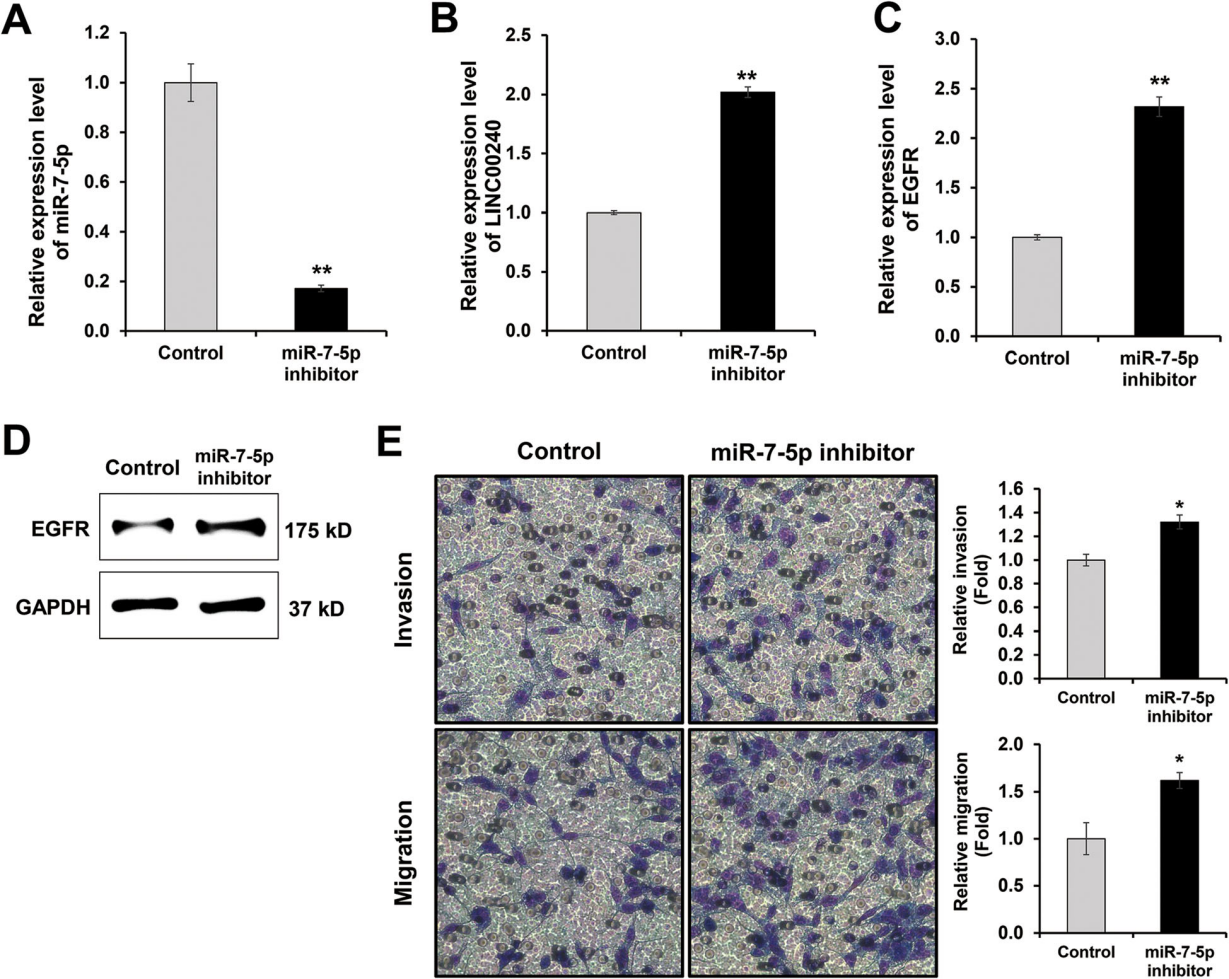
*miR-7-5p* suppresses cell invasion and migration in NSCLC. **a**-**d** Expression level of *miR-7-5p*, *LINC00240* and *EGFR* in Hcc1438 with inhibited *miR-7-5p*. **f** Inhibition of *miR-7-5p* increase cell invasion and migration in Hcc1438. *P < 0.05, **P < 0.01 and ***P < 0.001

Recent studies have shown that *miR-7-5p* suppressed cell invasion and migration in several cancers including colorectal, thyroid, colon, and gastric cancer [17–20]. However, the roles of *miR-7-5p* in NSCLC are not clearly understood. Thus, we examined the function of *miR-7-5p* in the regulation of cell motility in NSCLC. The cell invasion and migration ability of H1299 cells were decreased when *miR-7-5p* was overexpressed in H1299 cell (Fig. 1f). Next, we examined the knock-down effect of *miR-7-5p* expression in highly expressing miR-7-5p Hcc1438 cells (Fig. 2a). As expected, the expression levels of both *LINC00240* and *EGFR* were elevated in *miR-7-5p* silenced Hcc1438 cells (Fig. 2b, c, d). The loss of *miR-7-5p* also resulted in the increased cell invasion and migration ability of Hcc1438 cells (Fig. 2e). These findings demonstrated that *miR-7-5p* has a key role in the regulation of cell motility in NSCLC by regulating *EGFR* and *LINC00240* expression.

### Expression of *miR-7-5p* was regulated by *LINC00240*

**Fig. 3:**
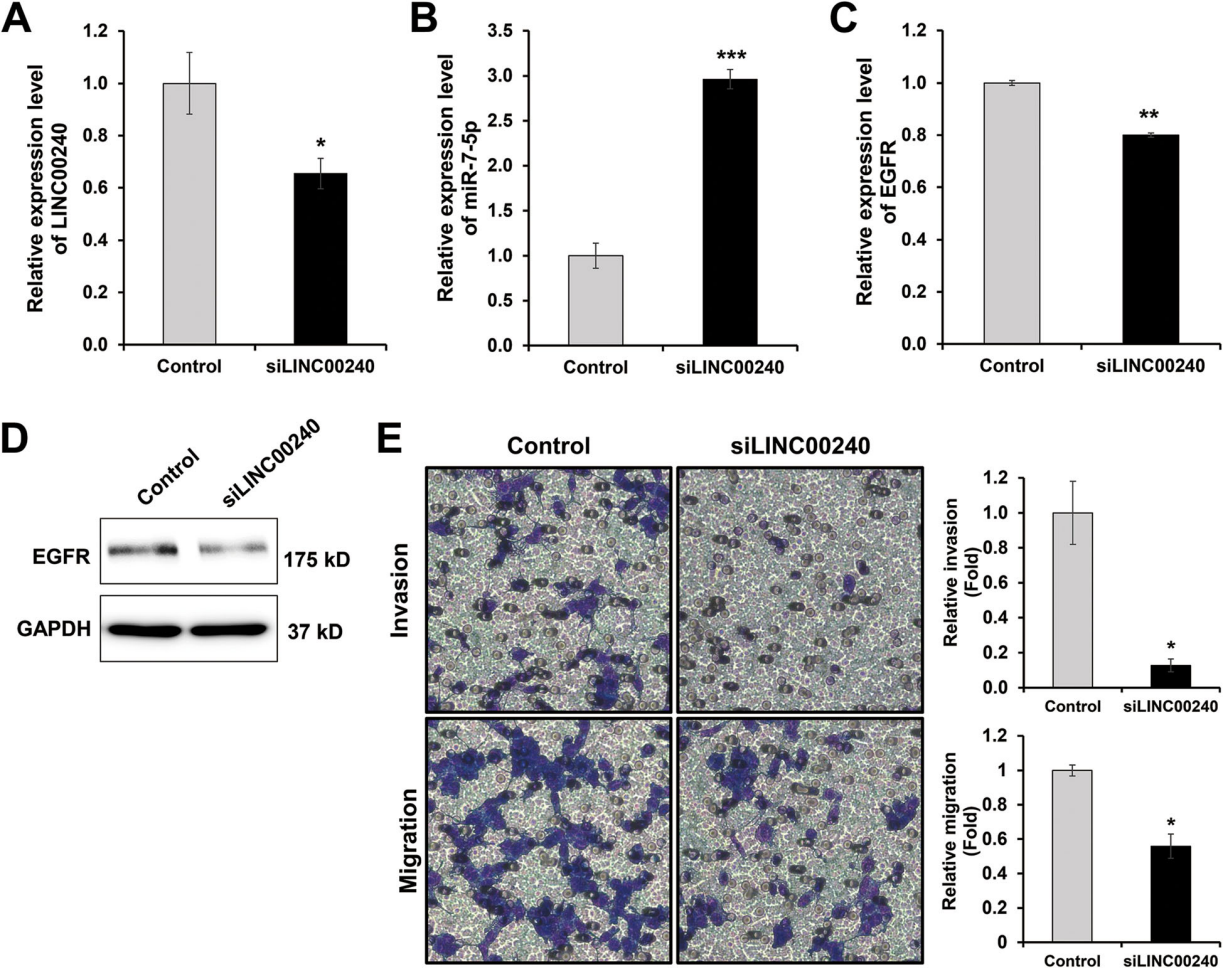
*LINC00240* regulates *miR-7-5p* and *EGFR* in NSCLC. **a**-**d** Expression level of *LINC00240*, *miR-7-5p* and *EGFR* in *LINC00240* knockdown H1299. **f** *LINC00240* knockdown decrease cell invasion and migration in H1299. *P < 0.05, **P < 0.01 and ***P < 0.001. siLINC00240: siRNA of *LINC00240*

To identify the relationship between *miR-7-5p* and *LINC00240*, we decreased the expression of *LINC00240* in the H1299 cells. Interestingly, the expression level of *miR-7-5p*, which may be a downstream regulator of *LINC00240*, was increased in *LINC00240*-silenced H1299 cells, whereas the *EGFR* expression was decreased compared to control cells (Fig. 3a-d). These results indicate that the expression network between *miR-7-5p* and *LINC00240* may involve a negative correlation. In the cell motility assay, *LINC00240* knock-down led to repressed cell invasion and migration in the H1299 cells (Fig. 3e). The phenotype was consistent with the results induced of *miR-7-5p* overexpression suggesting that *LINC00240* plays an important role in the cell invasion and migration process through the regulation of *miR-7-5p* expression.

### *LINC00240* as an *miR-7-5p* sponge regulator affected cell invasion and migration

**Fig. 4:**
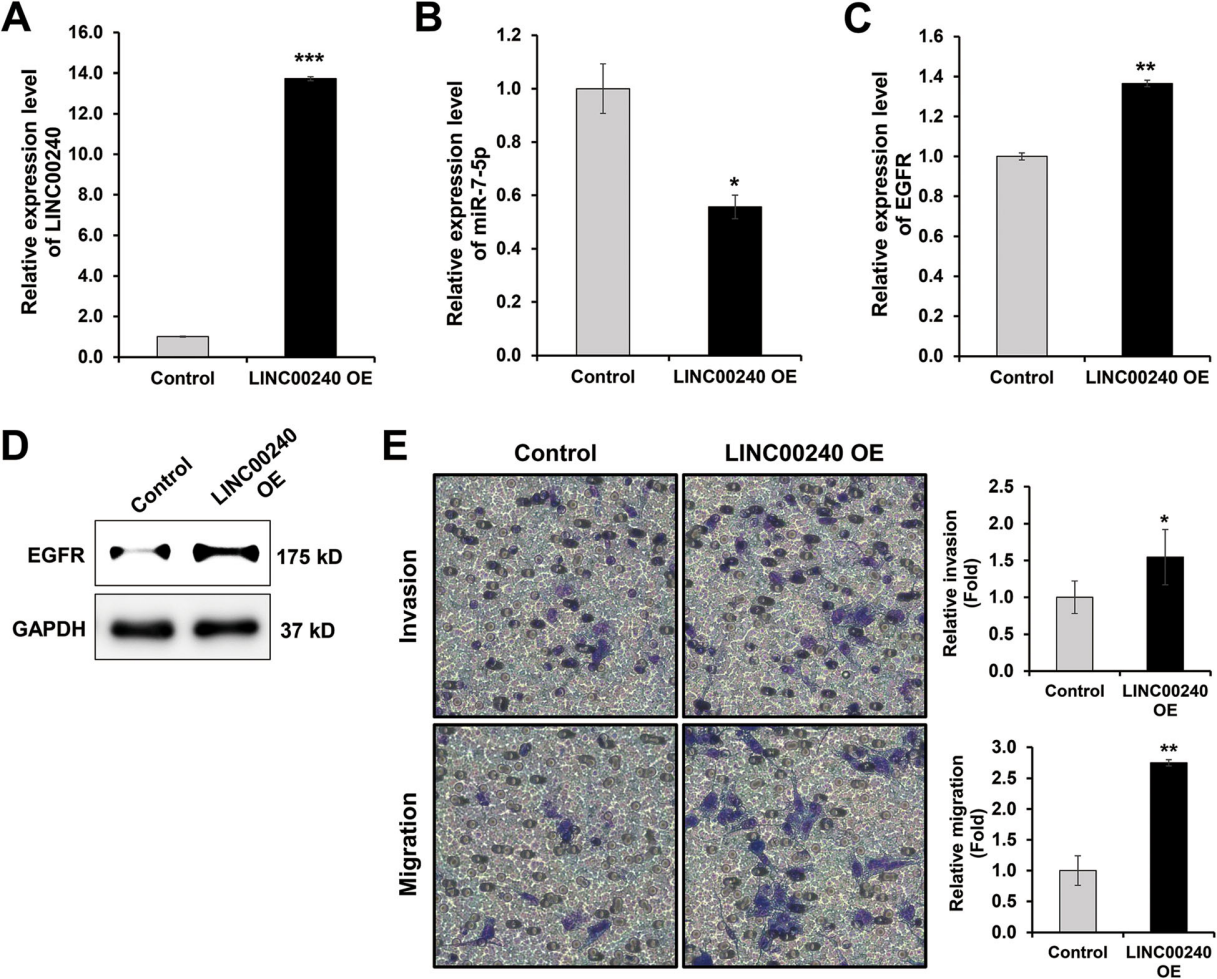
*LINC00240* regulates cell invasion and migration in NSCLC. **a**-**d** Expression level of *LINC00240*, *miR-7-5p* and *EGFR* in *LINC00240* overexpressed Hcc1438. **f** *LINC00240* overexpression increase cell invasion and migration in Hcc1438. *P < 0.05, **P < 0.01 and ***P < 0.001. LINC00240 OE: *LINC00240* overexpression

**Fig. 5:**
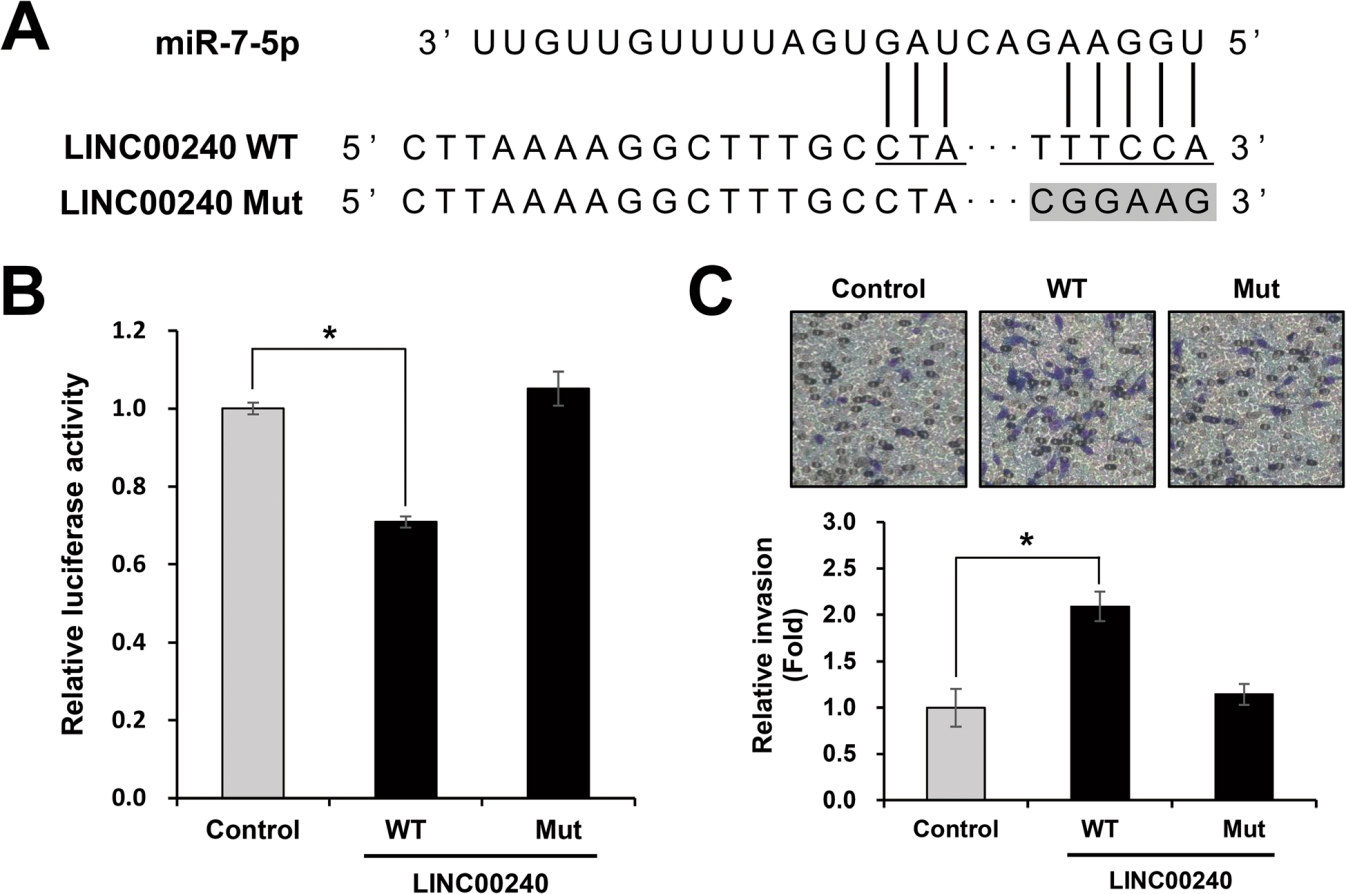
*LINC00240* directly interact *miR-7-5p*. **a** The sequence alignments of *miR-7-5p* and binding site with *miR-7-5p* within the *LINC00240*. **b**-**c** Hcc1438 were transfected with LINC00240-WT, and LINC00240-Mut containing the mutations of the *miR-7-5p* binding sites in the *LINC00240*. And the binding activity of *miR-7-5p* was measured using a luciferase assay and cell invasion. *P < 0.05. WT: Wild type; Mut: Mutant type

A recent study postulated that lncRNA plays a role as a negative regulator of miRNA expression in NSCLC [21]. So, we also examined the effect of *LINC00240* overexpression on the regulation of *miR-7-5p* expression. *LINC00240* overexpression decreased the expression level of *miR-7-5p* in the Hcc1438 cell (Fig. 4a). However, *EGFR* expression was upregulated ectopic expression *LINC00240* (Fig. 4b-d). In addition, the ectopic overexpression of *LINC00240* enhanced the cell invasion and migration capability of Hcc1438 cells (Fig. 4f). Moreover, Fig. 5 is shown that *LINC00240* directly interact with *miR-7-5p*. Luciferase activity was decreased in cell transfected of LINC00240-WT, whereas did not decrease in cell transfected of LINC00240-Mut containing the mutations of the *miR-7-5p* binding sites in the *LINC00240* (Fig. 5b). Also, cell invasion increased in LINC00240-WT transfected cell, but LINC00240-Mut did not affected cell invasion (Fig. 5c). These results may be related to the *LINC00240*-mediated *miR-7-5p* sponge regulatory network.

### *LINC00240* was up-regulated in lung squamous carcinoma

**Fig. 6:**
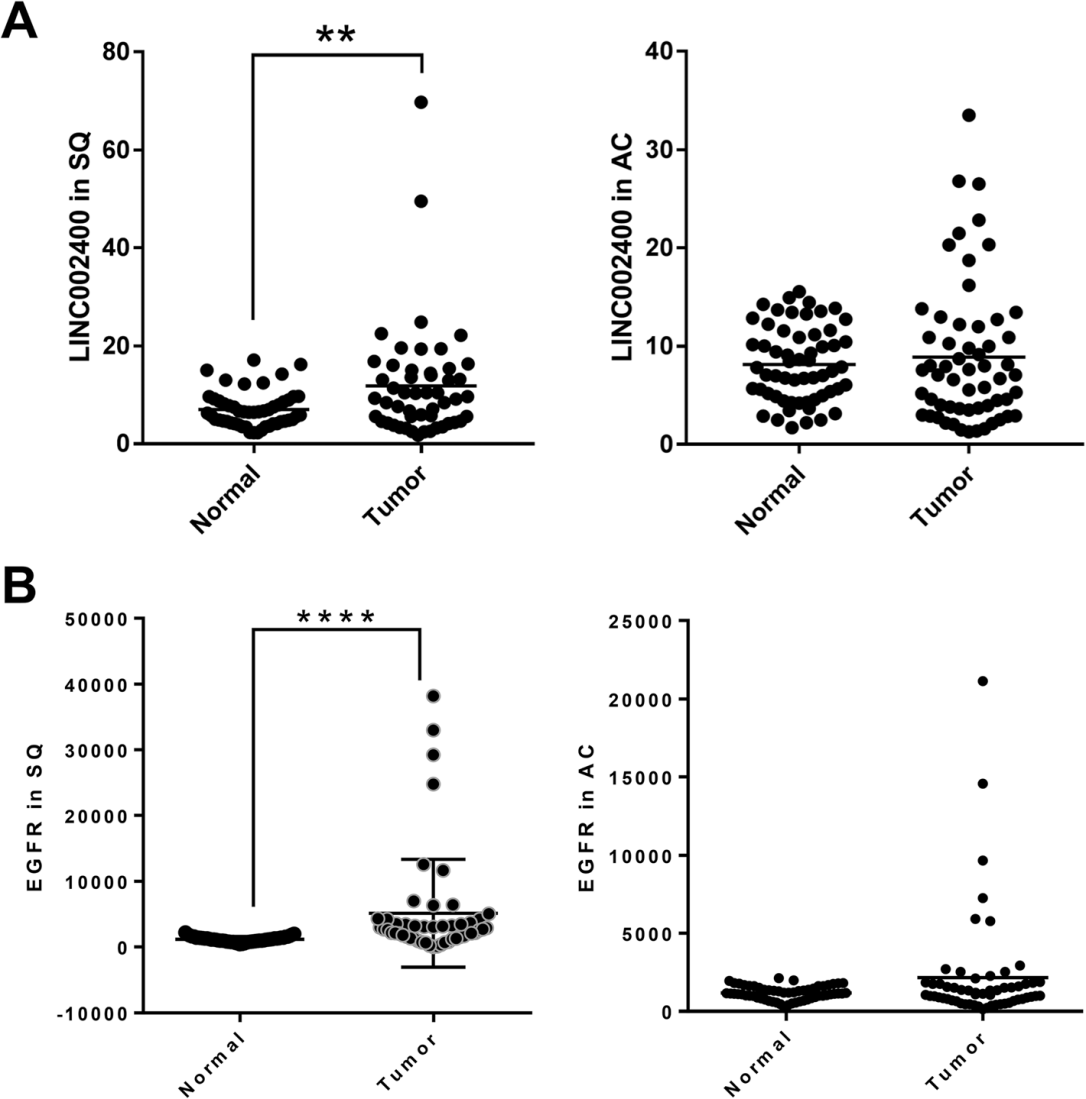
TCGA database analysis of LINC00240 (**a**) and EGFR (**b**) expression difference between tumor and normal tissues. **P < 0.01 and ****P < 0.0001. SQ: Squamous cell carcinoma; AD: Adenocarcinoma

We next examined the *LINC00240* expression in the TCGA data set of clinical lung cancers including lung adenocarcinoma and lung squamous carcinoma. As shown in Fig. 6a, *LINC00240* expression was significantly increased in the tumor tissue of the lung squamous carcinoma group compared to normal tissue. However, there was no statistically significant difference between lung adenocarcinoma and normal tissue in *LINC00240* expression. In addition, we examined the EGFR expression in the TCGA, as results of NSCLC cell, EGFR expression was significantly increased in the lung squamous carcinoma group (Fig. 6b). Therefore, these data suggest that the development and progression of lung squamous carcinoma were closely associated with the expression level of *LINC00240*.

## Discussion

We showed that *LINC00240* was downregulated in a lung cancer cell line after *miR-7-5p* mimic transfection. Further investigations revealed that the knockdown of *LINC00240* induced the upregulation of *miR-7-5p*. The overexpression of *miR-7-5p* diminished cancer invasion and migration. The *EGFR* expression was downregulated after *miR-7-5p* treatment. Silencing *LINC00240* suppressed the invasion and migration of the lung cancer cells, whereas *LINC00240* overexpression exerted an opposite effect. Furthermore, the lower expression of *LINC00240* in squamous lung cancer was analyzed using TCGA data.

Long noncoding RNAs (lncRNAs) are functionally defined as transcripts > 200 nt in length with no protein-coding potential, many of which are uniquely expressed in differentiated tissues or specific cancer types [22]. Long noncoding RNAs participate in the regulation of a variety of cell activities, such as cell differentiation, proliferation, invasion, apoptosis and autophagy, through interacting with RNAs, DNAs or proteins [23]. They play important roles in chromatin remodeling, transcriptional repression and post-transcriptional regulation [24]. It is now widely understood that lncRNAs serve as signals of specific cellular states or readouts of active cellular programs [25]. The molecular mechanisms of lncRNAs are traditionally classified into four archetypes: signals, decoys, guides and scaffolds. Several lncRNAs possess characteristics from multiple archetypes that, in combination, are critical to their eventual biological function. Recent studies revealed that some lncRNAs assumed the role of molecular sponges, a behavior akin to that of competitive endogenous RNAs (ceRNAs), through miRNAs binding sites, and subsequently repressed their inhibitory effect on their natural targets [4].

Moreover, lncRNAs have been observed to regulate complex cellular activities that are typically deregulated in cancer (e.g., cell growth, differential expression, and the maintenance of cell identity) [26]. The overexpression of the HOTAIR lncRNA in early-stage, surgically resected breast cancer was highly predictive of progression to metastatic disease and overall survival [27]. Subsequent studies elucidated the correlation between HOTAIR deregulation and cancer progression in 26 human tumor types [28]. By overlapping the cancer susceptibility loci determined by genome-wide association studies (GWAS), the presence of lncRNAs in specific tumors can also be examined. For instance, association analysis of the known risk loci certified through the genotyping of cancer patients elucidated the existence of a certain relationship between ANRIL, glioma, and basal cell carcinoma as well as an association of PTCSC3 with thyroid cancer [29, 30].

As small noncoding RNAs, microRNAs or miRNAs post-transcriptionally suppress cancer-related genes through attachment to the 3′-UTR of target mRNAs and thus act as oncogenes or tumor suppressor genes themselves [31]. MicroRNAs are involved in a range of processes that includes development, differentiation, proliferation, and apoptosis [32–34]. Multiple studies have shown that miRNAs play key roles in the metastasis of certain cancers, including gastric cancer, breast cancer, hepatocellular carcinoma, bladder cancer and lung cancer. Accumulating evidences has confirmed the indispensable role played by lncRNA sponges in cancer progression. Regarding lung cancer, the up regulation of a potent oncogene, ERBB4, generated by UCA1 was achieved by binding miR-193-3p [35]. In contrast, the sequestration of miR-181-a enabled MEG3 to up-regulate Bcl-2 in the case of gastric cancer [36]. In endometrial cancer stem cells, linc-RoR bound miR-145 in a similar manner, and ZFAS1 bound miR-150 in hepatocellular carcinoma. Likewise, CASC2 controlled the degree of miR-21 concentration [37]. Linc-RNA-RoR’s sponge-like behavior was determined to inhibit the miRNA-145 mediated differentiation of endometrial cancer stem cells [38]. Long non-coding RNA CASC2 suppressed malignancy in human gliomas by miR-21 [39]. The expansion of RNA-targeting therapeutics sheds new and unprecedented light on opportunities to efficiently modulate lncRNAs during potent anti-cancer therapies. While several strategies have been successfully employed to deplete lncRNAs, prior knowledge of lncRNA cellular localization is critical for selecting the appropriate strategy to achieve robust lncRNA modulation [40].

The role of *LINC00240* has been reported in esophageal squamous cell carcinoma (ESCC). The “loss” of miR-26b-5p-mediated *LINC00240*-KLF3 crosstalk was probably implicated in the tumorigenesis of ESCC [41]. *LINC00240* acts as an oncogene in cervical cancer progression by modulating the miR-124-3p/STAT3/MICA axis. The loss of *LINC00240* suppressed cervical cancer development through the sponging of miR-124-3p and the overexpression of *LINC0024*0 induced cervical cancer development. *LINC00240* expression promoted cervical cancer progression via the induction of miR-124-3p/STAT3/MICA-mediated NKT cell tolerance [42]. This study showed that the lncRNA of *LINC00240* sponged *miR-7-5p,* which is implicated in *EGFR* down-regulation.

Epidermal growth factor receptor (EGFR), a member of the Erb B receptor family, is widely expressed in human tissues and regulates important cellular processes, including proliferation, differentiation, and development. The establishment, growth and upkeep of epithelial tissues are primarily attributed to the EGFR signaling network, alterations in which may trigger malignant transformation [43]. One of the dysregulated miRNAs, *miR-7-5p*, has displayed its potential utility as a tumor suppressor in gastric cancers, breast cancers, and colorectal cancers [44–46]. *miR-7-5p* coordinately regulates EGFR signaling at multiple levels, suggesting that *miR-7-5p* additionally regulates a number of other cellular pathways relevant to normal and tumor cells and its ability to regulate oncogenic EGFR signaling in multiple cancer cell line models suggests that the therapeutic up-regulation of *miR-7-5p* expression in these tumors may inhibit growth and metastasis [16]. *miR-7-5p* suppresses the growth of lung cancer cells through others EGFR pathway. *miR-7* targets RAF1, IRS-2, BCL-2, and PA28γ in lung cancer cells. Besides EGFR pathway, *miR-7-5p* inhibits metastasis by targeting NOVA2 in NSCLC [47]. In addition, *miR-7* can increase the sensitivity of treatment resistant cancer cells to therapeutics and inhibit metastasis. These data suggest that replacement of *miR-7-5p* in specific human cancers could represent a new treatment approach [12].

While adenocarcinoma and squamous cell cancer are classified as NSCLC, these two carcinomas are distinguished from each other in terms of their respective occurrence at different anatomical sites as well as their molecular biologic background related to genetics and epigenetics. In the case of adenocarcinoma, in particular, driver mutations and the corresponding drugs already exists and thus, it reacts well to targeted therapy, which includes, but is not limited to EGFR, ALK, and ROS1, resulting in good outcomes. In contrast, appropriately targeted therapy drugs are yet to be identified for squamous cell cancer. *EGFR* overexpression or amplification occurs more frequently in squamous cell carcinoma than in adenocarcinoma, but *EGFR* mutations occurs mostly in adenocarcinoma. The significantly different expression of *LINC00240* only in squamous cell cancer provides clues to explaining the augmented *EGFR* expression, drawing on the findings of high expression of *EGFR* and marginal *EGFR* mutations in squamous cell cancer. In addition, the discovery of *EGFR* signaling pathway inhibition through lncRNA can shed light on the mechanism as a new potential target for lung cancer treatment.

## Conclusions

This research demonstrated that whereas *miR-7-5p* inhibited cancer growth and metastasis through the management of *EGFR*, *LINC00240* suppressed cancer metastasis by acting as a sponge for *miR-7-5p*. Consequently,LINC00240/miR-7-5p/EGFR axis may play important roles invasion and migration in NSCLC. As a single miRNA can inhibit a wide range of genes, it also has the capacity to regulate the expression of multiple oncogenes. Since lincRNA functions to control numerous miRNAs or genes, lincRNA has a high potential for tumor treatment.

## Supplementary Information

**Additional file 1: Table S1.** Total microarray data in miR-7-5p overexpression in H1299.

## Data Availability

The datasets generated during the current study are available in the Gene Expression Omnibus (GEO) repository (GSE158940).

## Abbreviations

LINC00240: Long intergenic non-protein coding RNA 240
lncRNA: long non-coding RNA
miRNA: micro RNA
ceRNA: competing endogenous RNA
EGFR: Epidermal growth factor receptor
NSCLC: Non-small cell lung cancer
AC: Adenocarcinoma
SQ: Lung squamous cell carcinoma
TCGA: The cancer genome atlas
UTR: Untranslated region
qRT-PCR: quantitative real time-polymerase chain reaction

## References

[1] Laskin JJ, Sandler AB (2005). State of the art in therapy for non-small cell lung cancer. Cancer Investig.

[2] Mercer TR, Dinger ME, Mattick JS (2009). Long non-coding RNAs: insights into functions. Nat Rev Genet.

[3] Kornienko AE, Guenzl PM, Barlow DP, Pauler FM (2013). Gene regulation by the act of long non-coding RNA transcription. BMC Biol.

[4] Tay Y, Rinn J, Pandolfi PP (2014). The multilayered complexity of ceRNA crosstalk and competition. Nature.

[5] Geisler S, Coller J (2013). RNA in unexpected places: long non-coding RNA functions in diverse cellular contexts. Nat Rev Mol Cell Biol.

[6] Ponting CP, Oliver PL, Reik W (2009). Evolution and functions of long noncoding RNAs. Cell.

[7] Wei M-M, Zhou G-B (2016). Long non-coding RNAs and their roles in non-small-cell lung cancer. Genomics Proteomics Bioinformatics.

[8] Xiong Y, Wang T, Wang M, Zhao J, Li X, Zhang Z, Zhou Y, Liu J, Jia L, Han Y (2018). Long non-coding RNAs function as novel predictors and targets of non-small cell lung cancer: a systematic review and meta-analysis. Oncotarget.

[9] Guo H, Ingolia NT, Weissman JS, Bartel DP (2010). Mammalian microRNAs predominantly act to decrease target mRNA levels. Nature.

[10] Deng Y, Deng H, Bi F, Liu J, Bemis LT, Norris D, Wang X-J, Zhang Q (2011). MicroRNA-137 targets carboxyl-terminal binding protein 1 in melanoma cell lines. Int J Biol Sci.

[11] Maas S (2010). Gene regulation through RNA editing. Discov Med.

[12] Kalinowski FC, Brown RA, Ganda C, Giles KM, Epis MR, Horsham J (2014). Leedman PJ: microRNA-7: a tumor suppressor miRNA with therapeutic potential. Int J Biochem Cell Biol.

[13] Kim JS, Song KS, Lee JK, Choi YC, Bang IS, Kang CS, Yu IJ (2012). Toxicogenomic comparison of multi-wall carbon nanotubes (MWCNTs) and asbestos. Arch Toxicol.

[14] Song KJ, Jeon SK, Moon SB, Park JS, Kim JS, Kim J, Kim S, An HJ, Ko JH, Kim YS (2017). Lectin from Sambucus sieboldiana abrogates the anoikis resistance of colon cancer cells conferred by N-acetylglucosaminyltransferase V during hematogenous metastasis. Oncotarget.

[15] Yu SL, Koo H, Lee HY, Yeom YI, Lee DC, Kang J (2019). Recombinant cell-permeable HOXA9 protein inhibits NSCLC cell migration and invasion. Cell Oncol.

[16] Webster RJ, Giles KM, Price KJ, Zhang PM, Mattick JS, Leedman PJ (2009). Regulation of epidermal growth factor receptor signaling in human cancer cells by microRNA-7. J Biol Chem.

[17] Qin A, Qian W (2018). MicroRNA-7 inhibits colorectal cancer cell proliferation, migration and invasion via TYRO3 and phosphoinositide 3-kinase/protein B kinase/mammalian target of rapamycin pathway suppression. Int J Mol Med.

[18] Xie J, Chen M, Zhou J, Mo MS, Zhu LH, Liu YP, Gui QJ, Zhang L, Li GQ (2014). miR-7 inhibits the invasion and metastasis of gastric cancer cells by suppressing epidermal growth factor receptor expression. Oncol Rep.

[19] Yue K, Wang X, Wu Y, Zhou X, He Q, Duan Y (2016). microRNA-7 regulates cell growth, migration and invasion via direct targeting of PAK1 in thyroid cancer. Mol Med Rep.

[20] Zeng CY, Zhan YS, Huang J, Chen YX (2016). MicroRNA7 suppresses human colon cancer invasion and proliferation by targeting the expression of focal adhesion kinase. Mol Med Rep.

[21] Sun C, Li S, Zhang F, Xi Y, Wang L, Bi Y, Li D (2016). Long non-coding RNA NEAT1 promotes non-small cell lung cancer progression through regulation of miR-377-3p-E2F3 pathway. Oncotarget.

[22] Iyer MK, Niknafs YS, Malik R, Singhal U, Sahu A, Hosono Y, Barrette TR, Prensner JR, Evans JR, Zhao S (2015). The landscape of long noncoding RNAs in the human transcriptome. Nat Genet.

[23] Lim LJ, Wong SY, Huang F, Lim S, Chong SS, Ooi LL, Kon OL, Lee CG (2019). Roles and regulation of long noncoding RNAs in hepatocellular carcinoma. Cancer Res.

[24] Derrien T, Johnson R, Bussotti G, Tanzer A, Djebali S, Tilgner H, Guernec G, Martin D, Merkel A, Knowles DG (2012). The GENCODE v7 catalog of human long noncoding RNAs: analysis of their gene structure, evolution, and expression. Genome Res.

[25] Wang KC, Chang HY (2011). Molecular mechanisms of long noncoding RNAs. Mol Cell.

[26] Hu W, Alvarez-Dominguez JR, Lodish HF (2012). Regulation of mammalian cell differentiation by long non-coding RNAs. EMBO Rep.

[27] Gupta RA, Shah N, Wang KC, Kim J, Horlings HM, Wong DJ, Tsai M-C, Hung T, Argani P, Rinn JL (2010). Long non-coding RNA HOTAIR reprograms chromatin state to promote cancer metastasis. Nature.

[28] Bhan A, Mandal SS (2015). LncRNA HOTAIR: a master regulator of chromatin dynamics and cancer. Biochim Biophys Acta.

[29] Jendrzejewski J, He H, Radomska HS, Li W, Tomsic J, Liyanarachchi S, Davuluri RV, Nagy R, De La Chapelle A (2012). The polymorphism rs944289 predisposes to papillary thyroid carcinoma through a large intergenic noncoding RNA gene of tumor suppressor type. Proc Natl Acad Sci.

[30] Pasmant E, Sabbagh A, Masliah-Planchon J, Ortonne N, Laurendeau I, Melin L, Ferkal S, Hernandez L, Leroy K, Valeyrie-Allanore L (2011). Role of noncoding RNA ANRIL in genesis of plexiform neurofibromas in neurofibromatosis type 1. J Natl Cancer Inst.

[31] Bartel DP (2004). MicroRNAs: genomics, biogenesis, mechanism, and function. Cell.

[32] Chen J-F, Mandel EM, Thomson JM, Wu Q, Callis TE, Hammond SM, Conlon FL, Wang D-Z (2006). The role of microRNA-1 and microRNA-133 in skeletal muscle proliferation and differentiation. Nat Genet.

[33] Cheng AM, Byrom MW, Shelton J, Ford LP (2005). Antisense inhibition of human miRNAs and indications for an involvement of miRNA in cell growth and apoptosis. Nucleic Acids Res.

[34] Zhang B, Pan X, Cobb GP, Anderson TA (2007). microRNAs as oncogenes and tumor suppressors. Dev Biol.

[35] Nie W, Ge HJ, Yang XQ, Sun X, Huang H, Tao X, Chen WS, Li B (2016). LncRNA-UCA1 exerts oncogenic functions in non-small cell lung cancer by targeting miR-193a-3p. Cancer Lett.

[36] Peng W, Si S, Zhang Q, Li C, Zhao F, Wang F, Yu J, Ma R (2015). Long non-coding RNA MEG3 functions as a competing endogenous RNA to regulate gastric cancer progression. J Exp Clin Cancer Res.

[37] Deng L, Yang S-B, Xu F-F, Zhang J-H (2015). Long noncoding RNA CCAT1 promotes hepatocellular carcinoma progression by functioning as let-7 sponge. J Exp Clin Cancer Res.

[38] Zhou X, Gao Q, Wang J, Zhang X, Liu K, Duan Z (2014). Linc-RNA-RoR acts as a “sponge” against mediation of the differentiation of endometrial cancer stem cells by microRNA-145. Gynecol Oncol.

[39] Wang P, Liu YH, Yao YL, Li Z, Li ZQ, Ma J, Xue YX (2015). Long non-coding RNA CASC2 suppresses malignancy in human gliomas by miR-21. Cell Signal.

[40] Lennox KA, Behlke MA (2016). Cellular localization of long non-coding RNAs affects silencing by RNAi more than by antisense oligonucleotides. Nucleic Acids Res.

[41] Yang S, Ning Q, Zhang G, Sun H, Wang Z, Li Y (2016). Construction of differential mRNA-lncRNA crosstalk networks based on ceRNA hypothesis uncover key roles of lncRNAs implicated in esophageal squamous cell carcinoma. Oncotarget.

[42] Zhang Y, Li X, Zhang J, Liang H (2020). Natural killer T cell cytotoxic activity in cervical cancer is facilitated by the LINC00240/microRNA-124-3p/STAT3/MICA axis. Cancer Lett.

[43] Yano S, Kondo K, Yamaguchi M, Richmond G, Hutchison M, Wakeling A, Averbuch S, Wadsworth P (2003). Distribution and function of EGFR in human tissue and the effect of EGFR tyrosine kinase inhibition. Anticancer Res.

[44] Li Y, Li Y, Liu Y, Xie P, Li F, Li G (2014). PAX6, a novel target of microRNA-7, promotes cellular proliferation and invasion in human colorectal cancer cells. Dig Dis Sci.

[45] Okuda H, Xing F, Pandey PR, Sharma S, Watabe M, Pai SK, Mo Y-Y, Iiizumi-Gairani M, Hirota S (2013). Liu Y: miR-7 suppresses brain metastasis of breast cancer stem-like cells by modulating KLF4. Cancer Res.

[46] Zhao X-D, Lu Y-Y, Guo H, Xie H-H, He L-J, Shen G-F, Zhou J-F, Li T, Hu S-J, Zhou L (2015). MicroRNA-7/NF-κB signaling regulatory feedback circuit regulates gastric carcinogenesis. J Cell Biol.

[47] Xiao H (2019). MiR-7-5p suppresses tumor metastasis of non-small cell lung cancer by targeting NOVA2. Cell Mol Biol Lett.

